# Stereotactic topography of the greater and third occipital nerves and its clinical implication

**DOI:** 10.1038/s41598-018-19249-6

**Published:** 2018-01-17

**Authors:** Hong-San Kim, Kang-Jae Shin, Jehoon O, Hyun-Jin Kwon, Minho Lee, Hun-Mu Yang

**Affiliations:** 1 Department of Anatomy, Yonsei University College of Medicine, Seoul, Korea; 2 Catholic Precision Medicine Research Center, College of Medicine, The Catholic University of Korea, Seoul, Korea; 3 Department of Anatomy, School of Medicine, Ewha Womans University, Seoul, Republic of Korea

## Abstract

This study aimed to provide topographic information of the greater occipital (GON) and third occipital (3ON) nerves, with the three-dimensional locations of their emerging points on the back muscles (60 sides, 30 cadavers) and their spatial relationship with muscle layers, using a 3D digitizer (Microscribe G2X, Immersion Corp, San Jose CA, USA). With reference to the external occipital protuberance (EOP), GON pierced the trapezius at a point 22.6 ± 7.4 mm lateral and 16.3 ± 5.9 mm inferior and the semispinalis capitis (SSC) at a point 13.1 ± 6.0 mm lateral and 27.7 ± 9.9 mm inferior. With the same reference, 3ON pierced, the trapezius at a point 12.9 ± 9.3 mm lateral and 44.2 ± 21.4 mm inferior, the splenius capitis at a point 10.0 ± 5.3 mm lateral and 59.2 ± 19.8 mm inferior, and SSC at a point 11.5 ± 9.9 mm lateral and 61.4 ± 15.3 mm inferior. Additionally, GON arose, winding up the obliquus capitis inferior, with the winding point located 52.3 ± 11.7 mm inferior to EOP and 30.2 ± 8.9 mm lateral to the midsagittal line. Knowing the course of GON and 3ON, from their emergence between vertebrae to the subcutaneous layer, is necessary for reliable nerve detection and precise analgesic injections. Moreover, stereotactic measurement using the 3D digitizer seems useful and accurate for neurovascular structure study.

## Introduction

On the posterior neck and occipital region, the posterior rami of the upper cervical spinal nerves issue occipital cutaneous nerves. The greater occipital nerve (GON) originates from the medial branches of the posterior rami of the second cervical spinal nerve. The nerve arises from intervertebral niche between the axis and atlas, and emerges from the suboccipital triangle. It travels along a route passing layers formed by the obliquus capitis inferior (OCI), semispinalis capitis (SSC), and trapezius, and arises on the subcutaneous layer, innervating the occipital area from the superior nuchal line (SNL) to the vertex. The medial branches of the posterior rami of the third cervical spinal nerve give off another smaller occipital cutaneous nerve, the third occipital nerve (3ON)^1–3^. The nerve arises from a space between the axis and the third cervical vertebra with or without a communication with GON. It innervates the skin on the lower part of the posterior neck.

These occipital cutaneous nerves are implicated in occipital neuralgia, in which paroxysmal shooting or stabbing pain occurs in the occipital region^4–7^. Pain in the posterior neck and occipital region can be caused by the compression or irritation of the occipital cutaneous nerves^5–9^. For instance, the entrapment of the occipital cutaneous nerves at the back muscles is considered a definitive cause of occipital neuralgia. In particular, GON is known to be frequently involved in occipital neuralgia^10^.

Local block anaesthesia of the occipital cutaneous nerves has been broadly implemented to diagnose or treat occipital neuralgia^11–14^. Hence, localizing the occipital cutaneous nerves is important for implementing analgesic procedures for occipital pain. Tenderness detection by palpation, stimulation of sensory nerves, recruitment of a Doppler flow probe, and ultrasound imaging have been used to localise the occipital cutaneous nerves^15–17^. Nevertheless, although clarifying the exact anatomical information regarding the occipital cutaneous nerves should help establish a diagnosis strategy aided by these tools, and improve the efficiency of block anaesthesia treatment, anatomical reports about the emergence and route of GON and 3ON are few, whereas, several studies have reported clinical phenomena after analgesic treatment for occipital neuralgia in living patients.

The overall course of GON and 3ON, from their emergence between vertebrae to the subcutaneous layer, should be elucidated for the efficient detection of the nerves and precise injection of analgesic agents. The emergence of the nerves from the back muscles is a useful landmark for the localization of these nerves. In addition, to estimate the diffusion of analgesic agents and to implement their efficient injection, a physician should consider the exact anatomy of the layers among the subcutaneous tissue, trapezius, splenius capitis (SpC), SSC, and OCI muscles^17^. Furthermore, the relative position of the occipital cutaneous nerves with layers formed by these back muscles can provide useful information for the interpretation of the sonographic image during ultrasonic-guided injection^1^.

Hence, the present study aimed to provide comprehensive topographic information of GON and 3ON with the three-dimensional (3D) locations of their emerging points on the back muscles (60 sides of 30 cadavers), and their topographic relationship with layers formed by the muscles by means of a 3D digitizer. We also provided 3D supplementary images showing metric information of the two occipital cutaneous nerves to facilitate the understanding of their stereoscopic topography.

## Results

### Emergences of the occipital cutaneous nerves from the trapezius muscle

**Figure 1:**
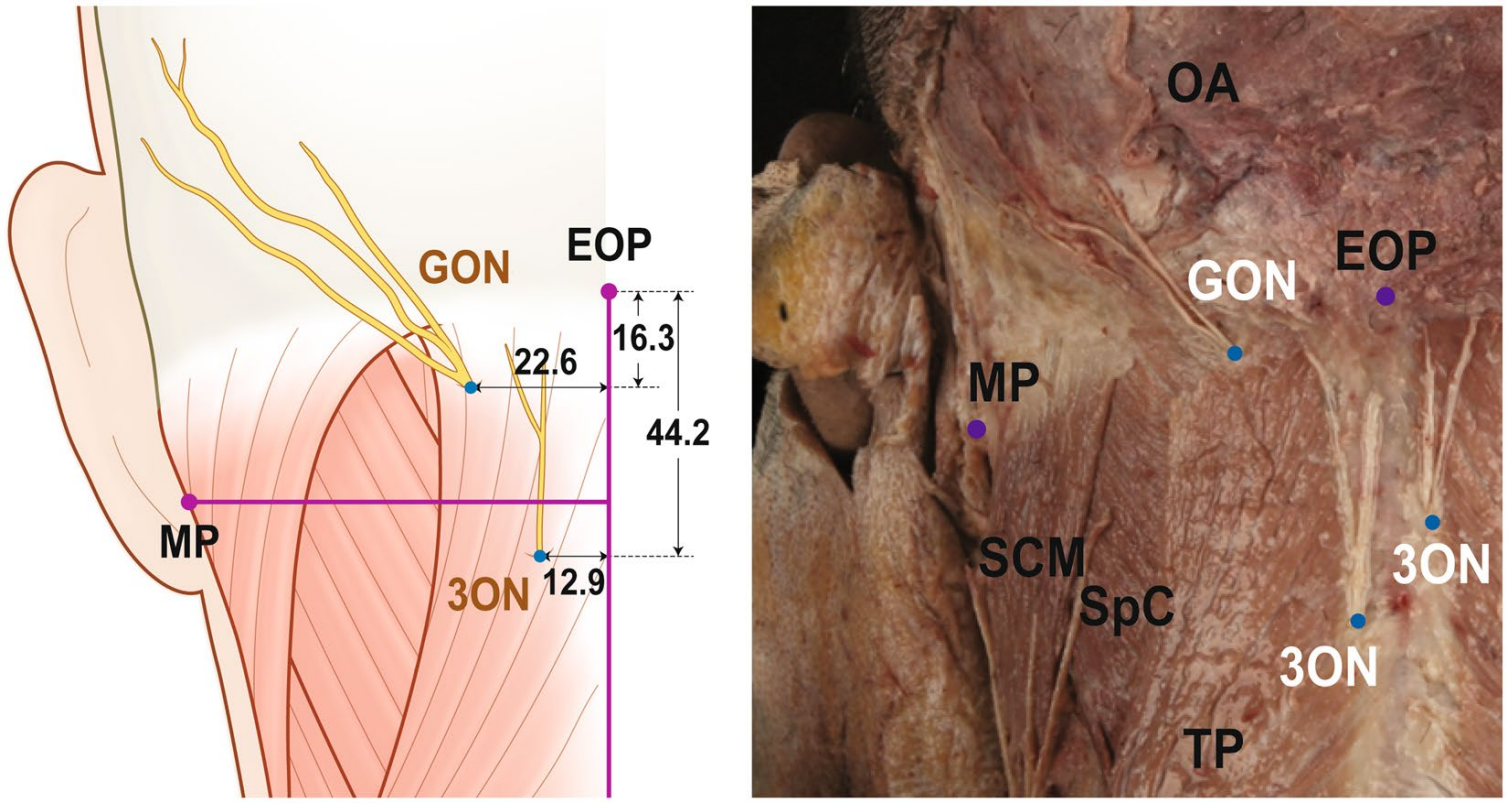
The locations of emergences of greater occipital nerve (GON) and third occipital nerve (3ON) from the trapezius muscle (TP). The blue dots indicate the emergences of nerves. external occipital protuberance, EOP; sternocleidomastoid muscle, SCM; splenius capitis, SpC; mastoid process (lowermost point), MP; occipital artery, OA. The numbers in table are presented as mean ± SD in millimetre.

GON and 3ON emerged from the trapezius muscle after piercing the muscle. The emergences of the two occipital cutaneous nerves from the trapezius muscle are shown in Fig. 1 and Table 1 with metric information. GON pierced the trapezius muscle near an aponeurotic portion of the trapezius attached to SNL. At the vicinity of the emergence of GON, there was a piercing point of the occipital artery (OA) to the trapezius. Horizontally arranged aponeurotic fibres of the trapezius muscle tendon lay below the piercing points of GON and OA.

**Table 1:** The location of emergences of greater occipital nerve (GON) and third occipital nerve (3ON) from the trapezius muscle.

	Horizontal distance from Midline (mm)	Vertical distance from external occipital protuberance (mm)
Emergence of GON on the Trapezius	22.6 ± 7.4	16.3 ± 5.9
Emergence of 3ON on the Trapezius	12.9 ± 9.3	44.2 ± 21.4

The piercing point of GON was 22.6 (mean) ± 7.4 (SD) mm lateral to the midline and 16.3 ± 5.9 mm inferior to the external occipital protuberance (EOP). After emerging from the trapezius muscle, GON was divided into several branches that proceeded superolaterally and dispersed in the occipital region.

3ON also emerged from the trapezius muscle after piercing it at a point 12.9 ± 9.3 mm lateral to the midline and 44.2 ± 21.4 mm inferior to EOP. After emerging from the trapezius, 3ON was divided into 1–3 ascending branches which nearly reached SNL.

The distance between the emerging points of GON and 3ON on the trapezius muscle was 44.4 mm on average.

### Emergences of the occipital cutaneous nerves from the splenius capitis and semispinalis capitis

GON pierced SSC at a point 13.1 ± 6.0 mm lateral to the midline and 27.7 ± 9.9 mm inferior to EOP, and did not predominantly pierce SpC.

The piercing point of GON in SSC was situated within 3 cm from the midline in all cases and within 4 cm from EOP in 91.7% (55/60) of cases. After piercing SSC, GON ascended laterally toward its emergence point on the trapezius muscle.

3ON pierced SpC at a point 10.0 ± 5.3 mm lateral to the midline and 59.2 ± 19.8 mm inferior to EOP. This nerve also pierced SSC at a point 11.5 ± 9.9 mm lateral to the midline and 61.4 ± 15.3 mm inferior to EOP. The piercing points of 3ON in SpC and SSC were very close to each other. The piercing point of 3ON into SSC was located within 1.5 cm from the midline in 86.7% (52/60) and below 4 cm from EOP in 76.7% (46/60) of cases.

**Figure 2:**
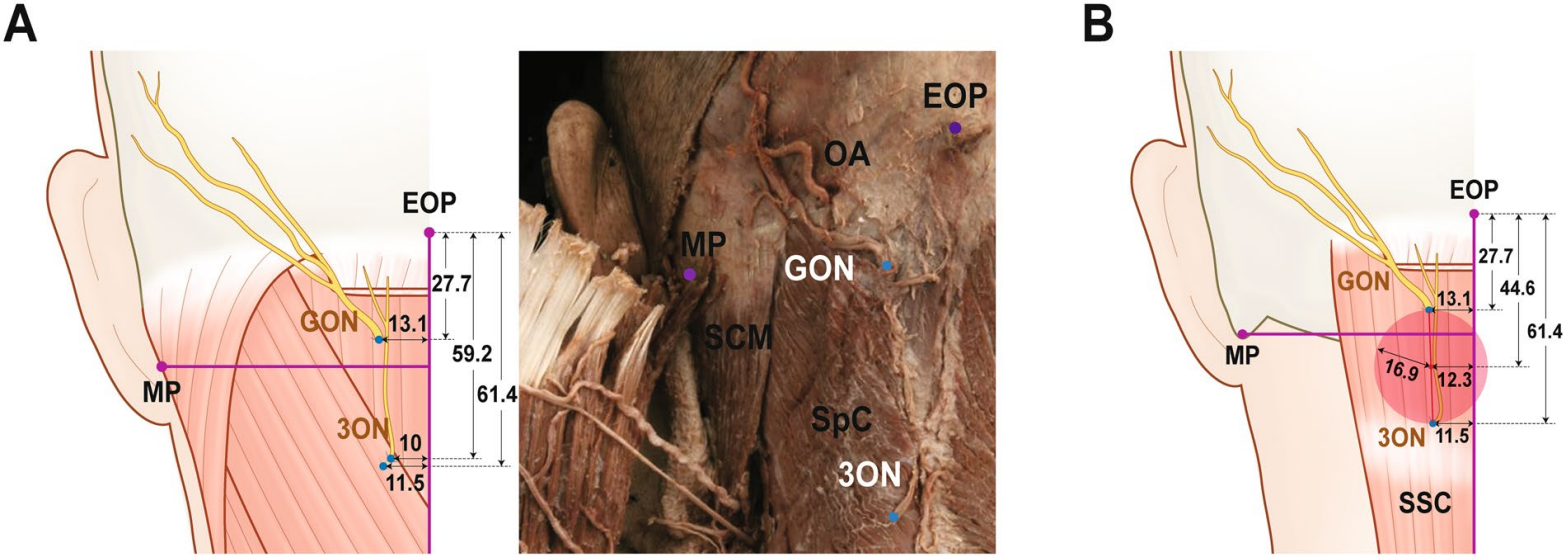
The locations of emergences of greater occipital nerve (GON) and third occipital nerve (3ON) from the semispinalis capitis (SSC) and splenius capitis (SpC). (**A**). Locations of emergences (blue dots) of two nerves from SSC and SpC. The emergence of 3ON from the SSC is covered by the SpC. (**B**). Midpoint (red dot) of emergences (blue dots) of two nerves from the SSC. A reddish circle means the area within the mean distance between the midpoint and two emergences. external occipital protuberance, EOP; sternocleidomastoid muscle, SCM; mastoid process (lowermost point), MP; occipital artery, OA. All numbers are presented in millimetre and the numbers in table are presented as mean ± SD.

The emergences of the two occipital cutaneous nerves were both observed on a plane above SSC. At this plane, the midpoint of the two emerging points was located 12.3 ± 6.4 mm lateral to the midline and 44.6 ± 10.8 mm inferior to the EOP. The distance between the midpoint and two emerging points was 16.9 mm. All the aforementioned descriptions are shown in Fig. 2. and Table 2.

**Table 2:** The locations of emergences of greater occipital nerve (GON) and third occipital nerve (3ON) from the semispinalis capitis and splenius capitis.

	Horizontal distance from Midline (mm)	Vertical distance from external occipital protuberance (mm)
Emergence of GON on the Semispinalis Capitis	13.1 ± 6.0	27.7 ± 9.9
Emergence of 3ON on the Splenius Capitis	10.0 ± 5.3	59.2 ± 19.8
Emergence of 3ON on the Semispinalis Capitis	11.5 ± 9.9	61.4 ± 15.3

### Position of the greater occipital nerve winding around the obliquus capitis inferior

**Figure 3:**
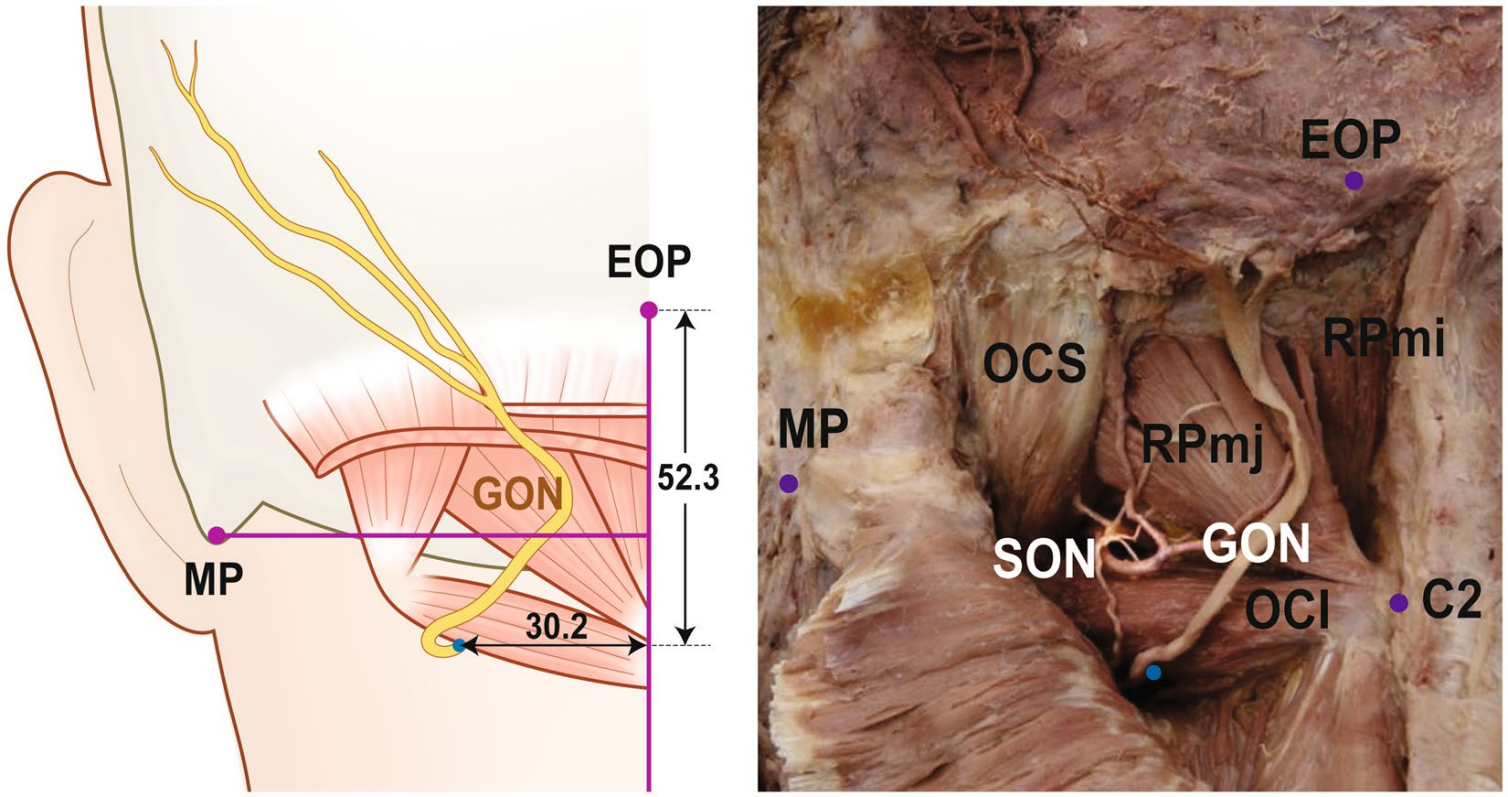
The location of winding point (blue dots) of greater occipital nerve (GON) on obliquus capitis inferior (OCI). external occipital protuberance, EOP; mastoid process (lowermost point), MP; obliquus capitis superior, OCS; rectus capitis posterior major, RPmj; rectus capitis posterior minor, RPmi; suboccipital nerve, SON; tip of spinous process of the axis, C2. All numbers are presented in millimetre and the numbers in table are presented as mean ± SD.

OCI, an intrinsic back muscle lying deep to SSC and the trapezius muscles, was also established as a landmark structure in the present study (Fig. 3 and Table 3). The muscle originates from the spinous process of the axis, proceeds superolaterally, and is inserted into the transverse process of the atlas. GON arose winding up OCI and the winding point was located 52.3 ± 11.7 mm inferior to EOP and 30.2 ± 8.9 mm lateral to the midsagittal line. The nerve then ascended between OCI and SSC before piercing SSC.

**Table 3:** The location of winding point of greater occipital nerve (GON) on obliquus capitis inferior.

	Horizontal distance from Midline (mm)	Vertical distance from EOP (mm)
Position of GON winding around the Obliquus Capitis Inferior	30.2 ± 8.9	52.3 ± 11.7

**Figure 4:**
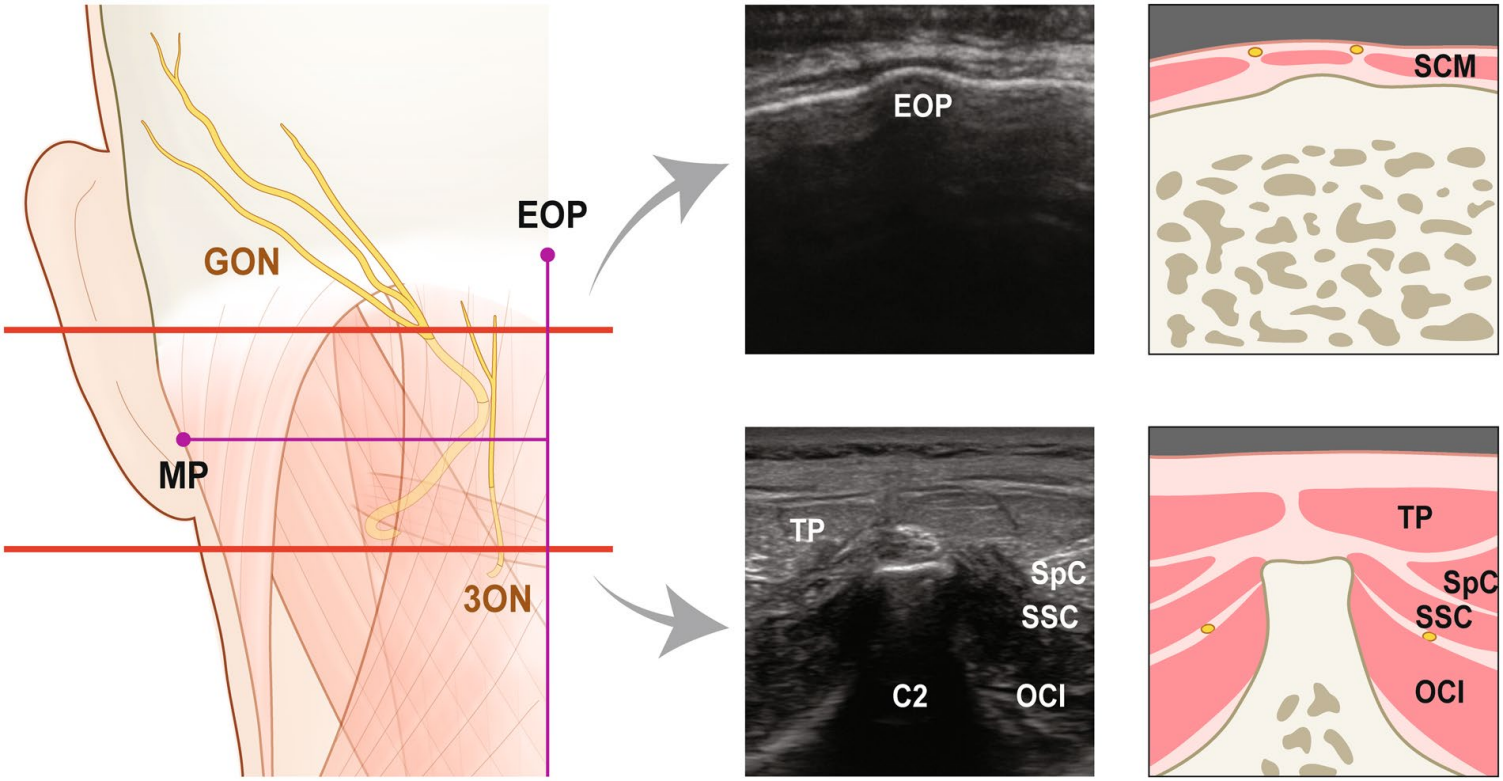
The position of greater occipital nerve (GON) and third occipital nerve (3ON) in cadaveric observation (left illustration) and the corresponding ultrasound images (right images). Upper red line runs through the emergence of GON from the trapezius (TP) and lower one through the spinous process of the axis (C2). Each ultrasound images and their illustrations corresponds to sections through upper and lower red lines respectively. sternocleidomastoid muscle, SCM; splenius capitis, SpC; semispinalis capitis, SSC; obliquus capitis inferior, OCI.

On an ultrasound image taken at the level of the axial spinous process, the position of GON corresponded with that observed in our study (Fig. 4). The nerve was situated between OCI and SSC.

### Overall course of the occipital cutaneous nerves

**Figure 5:**
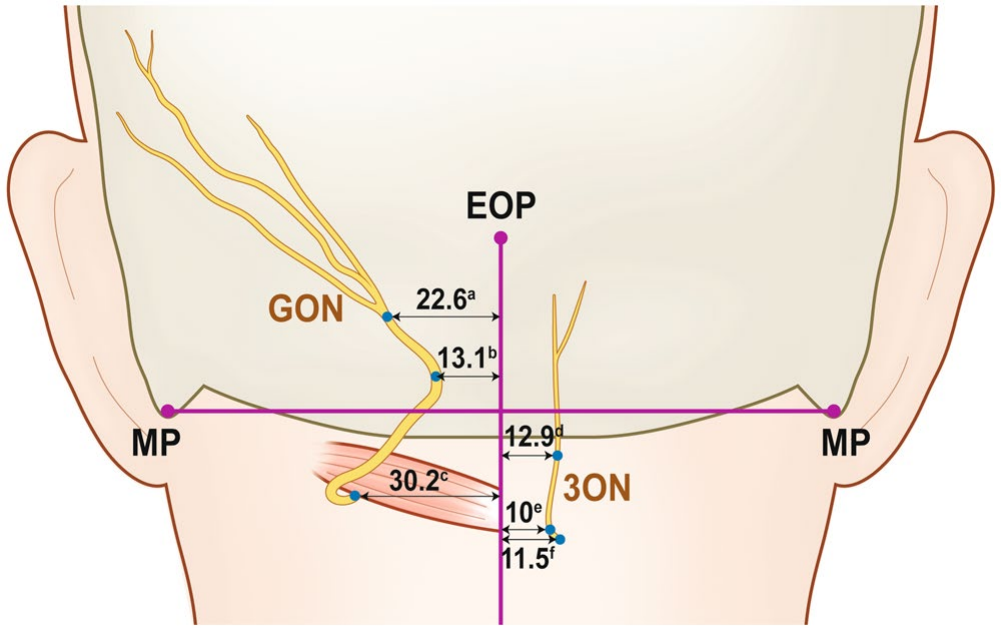
The overall course of the greater occipital nerve (GON) and third occipital nerve (3ON) with horizontal distances (arrows) between their emergences (a~f) from the back muscles and the midline. The blue dots mean the emergences of GON from trapezius (a), semispinalis capitis (b) and occipital capitis inferior (c), and the emergences of 3ON from trapezius (d), splenius capitis (e) and semispinalis capitis (f). All numbers are presented in millimetre. external occipital protuberance, EOP; mastoid process (lowermost point), MP.

**Figure 6:**
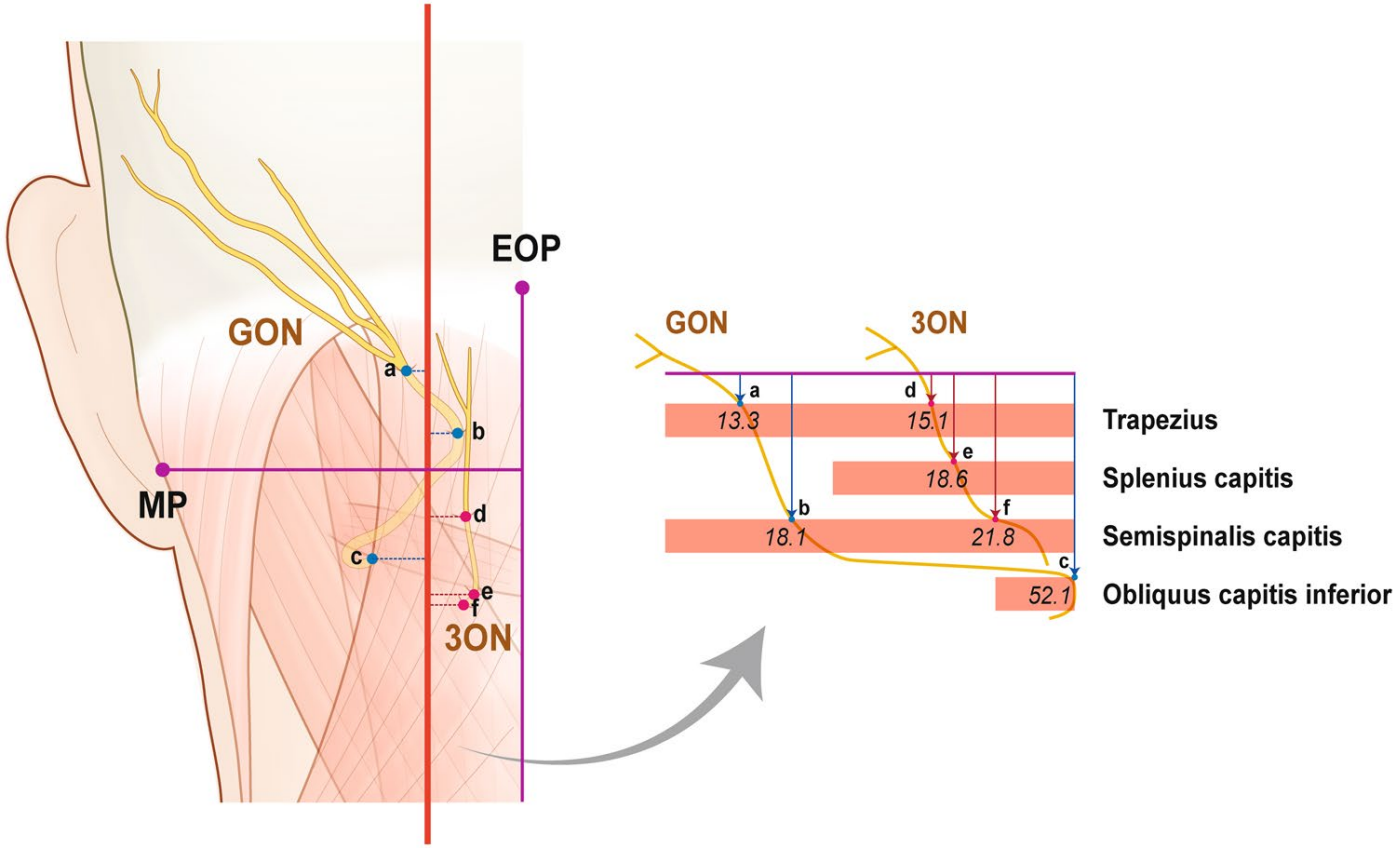
The depth of emergences of the greater occipital nerve (GON) and third occipital nerve (3ON) from the back muscles. The reference line (a violet line in the right illustration) is established a line from the external occipital protuberance (EOP) to the vertebra prominens (7^th^ cervical vertebra). The blue dots mean the emergences of GON from trapezius (a), semispinalis capitis (b) and occipital capitis inferior (c). The red dots mean the emergences of 3ON from trapezius (d), splenius capitis (e) and semispinalis capitis (f). Each indicating arrow means the depth of the emergence from the reference line. All numbers are presented in millimetre. Mastoid process (lowermost point), MP.

Since GON ascended medially, its relative position with the midsagittal line varied according to the various levels. The overall course of the two occipital cutaneous nerves may be seen in Fig. 5 and the depth of the piercing points of GON and 3ON are shown in Fig. 6.

Mostly, from the inferior border of OCI to the insertion part in SSC, GON proceeded beneath SSC. The nerve notably changed its course at this layer; it ascended vertically after winding around OCI and then turned medially at the upper border of this muscle, close to the midsagittal line. Before piercing SSC, GON ascended vertically, approaching the midsagittal line. It changed its course laterally, proceeding superolaterally before emerging from the trapezius muscle.

3ON ascended vertically piercing SSC, SpC, and the trapezius muscle serially. The emergence of this nerve from the trapezius was at a point 43.3 mm inferior to that of GON.

There were several unilateral communications between GON and 3ON, whereas, there were no bilateral communications among the cutaneous nerves because of the ligamentum nuchae at the midsagittal plane.

## Discussion

Generally, the posterior rami of the spinal nerves divide into medial and lateral branches that innervate the muscles and skin of the posterior neck and trunk. The posterior ramus of the second cervical spinal nerve divides into a medial and lateral branch below OCI; the lateral branch is a larger motor nerve supplying the longissimus capitis, SSC, and SpC, and the medial branch is a larger sensory nerve innervating the scalp skin as far forward as the bregma^18^. This larger sensory nerve, GON, ascends with OA across OCI, SSC, and the trapezius muscle and emerges from the subcutaneous layer of the posterior neck. GON is a distinct nerve including the second cervical spinal nerve, which communicates with the first cervical spinal nerve (suboccipital nerve), third, or lesser occipital nerve during its entire course.

The piercing position of GON is clinically important for the diagnosis and treatment of pain syndrome in the posterior neck region. Regarding the several muscular layers at the back region, the layered topography of the nerve should be known for efficient block anaesthesia on the back. In our study, GON pierced the trapezius muscle about 16 mm inferior to EOP and 22 mm lateral to the midline. Previous studies also described GON with reference to the midsagittal line, SNL, or EOP. Previous studies reported that GON pierced the trapezius at a point about 1–2.5 cm inferior to EOP and 1.5–3.7 cm lateral to the midsagittal line^4,7,19^. Mosser *et al*. reported that GON was located 1.5 cm lateral to the midsagittal line and 3 cm below EOP and Natsis *et al*. reported that GON was located 1.2 cm lateral to the midline and 27 mm below EOP^2,8^. Likewise, a variability in the horizontal distance of GON from the midline was shown in the present study.

The layer between OCI and SSC is a space where GON and 3ON both emerge. In the present study, GON emerged from OCI about 3 cm lateral to the midline and 5 cm deep to the skin. Although the thickness of the subcutaneous layer at the back varies among individuals, the depth of emergence of the nerve on OCI is relatively constant. Mostly, 3ON is located medial to the emergence of GON at this layer. Thus, a diffusion of agents medial to this emergence point can enhance the anaesthetic effect on not only GON but also 3ON. Given that the diffusion area of anaesthetic agents can compensate their positional deviation, this location can be regarded as an injection target.

Occipital neuralgia is a well-known pain syndrome with paroxysmal shooting pain in the dermatome of the second cervical spinal nerve^4^. Generally, the pain begins in the suboccipital region and spreads throughout the upper neck, back of the head, and vertex^20^. The causes of pain in the occipital region could be vascular (dural arteriovenous fistula, bleeding from bulbocervical cavernomas, or giant cell arteritis), neurogenic (schwannoma at the craniocervical junction, C2 myelitis, or multiple sclerosis), or osteogenic (C1/C3 arthrosis, hypermobile C1 posterior arch, or cervical osteochondroma). One popular aetiology of occipital neuralgia is an entrapment of the occipital cutaneous nerves at the points where they pierce the back muscles^3^. The most common trigger of occipital neuralgia is the compression of GON and lesser occipital nerve. Although the lesser occipital nerve is also one of the occipital cutaneous nerves, GON has a higher frequency of involvement in the pain symptom (90% vs 10%)^4^.

Previously, GON has been described as piercing the back muscles along its length. These muscular investments could entrap the nerves and serve as a source of nerve compression. In the present study, the main trunk of GON could be entrapped at its piercing point on the trapezius and SSC. However, whereas Bovim *et al*. reported that GON pierced OCI in 7.5%, the main trunk was not entrapped within this muscle in our study^19^. Instead, several small branches, which were ramified from the main trunk, innervated OCI. Notably, we found the transverse fibres below the piercing point of GON into the trapezius in most cases. This region belonged to the insertion part of the trapezius and most of the muscular belly was arranged vertically. Recently, Watanabe *et al*. reported that the transversus nuchae, one of the muscles comprising the superficial musculoaponeurotic system, originated from the fascia surrounding the EOP and proceeded inferolaterally towards the mastoid process in about 30%^21^. This muscle had 1–3 bundles that ran below the sling where GON pierced. Although, the transverse fibers below the piercing point of GON were not shown as distinct muscle fibers in our study, a possibility of the contraction of the transversus nuchae as a trigger of occipital neuralgia cannot be excluded. The anatomical feature of GON and its piercing points in the present study supports early evidence suggesting a peripheral mechanism for headaches by the facial or muscular entrapment of the nerve.

The exact stereotactic topography of GON and 3ON and their overall course in the present study can help in block anaesthesia treatment or open surgery for pain syndrome with ultrasonography. In particular, the injection of local anaesthetic agents can result in a transient effect or even long-term pain subsidence^4,22,23^. Various injection points have been recommended. One of the suggested target points is the piercing point of GON into the trapezius, 1.5–2 cm lateral to the midline and 2–2.5 cm inferior to EOP^7,24–28^. Actually, the level 2 cm below EOP might be a reasonable target area considering that GON ascends after piercing. However, Greher *et al*. recommended the C2 level (inferior border of the suboccipital triangle), where GON loops OCI^17^. They reported a block success rate of 80% near SNL and 100% near the C2 level. GON in this region is more proximal and 3ON is also closely situated. Moreover, more variability in number and position was shown at the level of EOP than at the C2 level.

Unlike other previous studies, the present study provided two additional considerations for clinical application; the depth of the cutaneous nerves and their overall routing course. To our knowledge, the depth of the nerves has been unclear to date. The layered structure of the back muscles is important for estimating the diffusion of injected substances. The layer between the trapezius and SSC near SNL and the suboccipital triangle beneath SSC at the C2 level have been commonly recruited as target areas for block procedures. At this plane, the location 12 mm lateral to the midline and 45 mm inferior to EOP enables the spread of analgesic agent to both piercing points of GON and 3ON into SSC. This layer is a thin potential space packed with subcutaneous tissue and, hence, a small amount of agent could penetrate this layer to simultaneously block the two cutaneous nerves.

The depth of the piercing points of GON and 3ON into SSC is about 2 cm. With ultrasonography clarifying the layered structure of this region, this anatomical information can help to properly estimate the injection site. Regarding the variable appearance of OCI based on to the angle of the ultrasound sensor, the fact that GON loops OCI at a location 3 cm lateral to the midline can help the physician target the looping position of the nerve.

3ON emerged almost below the emergence of GON from SSC in most cases in our study. Before emerging from the trapezius muscle below GON, 3ON could pierce SpC after passing through SSC. For diagnostic injections, the piercing point of 3ON into SpC, which was 1 cm lateral to the midline and 6 cm below the EOP, could serve as a useful landmark since GON did not pierce SpC. GON ascended almost perpendicularly after looping OCI, and turned medially below the rectus capitis major. The nerve ascended vertically on this muscle very close to the midline and furcated into several branches that proceeded superolaterally after piercing the trapezius. The fact that the bilateral GON approached each other very closely at about the C1 level can provide useful information for identifying the nerve via ultrasound. However, an injection into the midline at this level cannot block both GONs because of the ligamentum nuchae. Instead, the bilateral points slightly lateral to the midline at the C1 level can be efficient injection points and these points might be easily detected on ultrasonography.

Traditional anatomical studies have performed direct measurement using digital calipers or two-dimensional (2D) morphological analysis using photographs for gathering information on the location and dimensions of specific neurovascular structures^29^. Recently, 3D geometric analysis has been performed using computed tomography (CT) image analysis or a 3D digitizer such as Microscribe G2X (Immersion Corp, San Jose CA, USA)^29–31^. The CT image is very useful for measuring the anatomical structure of both living persons and cadavers. For instance, Mottini *et al*. reported the stereographic information of orbital volume using a digital reproducible evaluation method with 3D CT^32^. However, CT image analysis is limited regarding the detection of the exact location of a structure because of insufficient resolution, and it has low practical accessibility for observing the whole cadaver. However, the Microscibe 3D digitizer is a portable measurement tool that can easily determine exact coordination data and, thus, can be used to evaluate the stereotactic topography of anatomical structures in a cadaver. Coordinates taken by the 3D digitizer can be used to determine the distance between two landmarks, length of a structure, angles of three points, or extent of some areas. Hence, the Microscribe 3D digitizer has been used to collect biological anthropology information over the recent last decades^29^. In the clinical field, cadaveric studies using this 3D digitizer were performed for 3D geometry evaluations of bony structures such as the scapula and humerus^30,31^. However, stereotactic measurement with a 3D digitizer has hardly been applied to soft tissue structures. In our study, the structures of the suboccipital region were too complicated for 2D linear measurement using a digital caliper. We acquired precise stereotactic information of GON and 3ON and landmark structures using the 3D digitizer. In particular, the depth position (z-axis) of two nerves could be easily determined, and the coordinates were used in the reconstructive process of their entire course.

In stereotactic coordinate analysis, the distance between two points can be simply calculated using the Pythagorean Theorem and many anthropologic studies performed simple calculations to determine the distance between two points. The distance between a reference plane and a specific point can be determined after the set-up of the reference plane. We created a program to analyse coordinates (midplane/coronal reference plane set-up and distance determination procedure). Our algorithm can be applied to biological anthropology; it can be used to analyse bilateral asymmetry more accurately.

We also provided a supplementary file regarding the 3D information. This digital information could also be useful clinically.

This study has some limitations that need to be considered. We excluded subjects with severe asymmetry in abnormal positions. Like all other anatomy studies, the observed area of the cadavers used in this study seemed slightly asymmetric and, thus, differences in contraction of bilateral muscles could have affected the position of piercing points of the nerves in the muscle. The midplane, a reference plane in our study, was defined as a plane through three points – EOP, the spinous process of the vertebra prominens, and the midpoint between the bilateral lowermost points of the mastoid process. Although there is a difference between the ideal midsagittal plane and our midplane, we considered it insignificant. The depth taken, with reference to the line through EOP and the vertebra prominens, may not perfectly correspond to the real depth from the skin.

To conclude, the anatomical information of GON and 3ON and their relationship with the back muscles in the present study is useful for performing a nerve block for occipital neuralgia with clear landmarks such as EOP, the mastoid process, and midline. Stereotactic information can help a physician establish an efficient injection plane and estimate the spread of agents with ultrasound imaging. A plane between the trapezius and SSC and the area around the transverse process of the axis may be considered reasonable targets for the efficient nerve block of the two cutaneous nerves simultaneously. GON can be easily manipulated near SNL and the C2 level. The injection of a small amount of agent into a layer between SSC and SpC can be used for differential diagnosis. In addition, we showed that stereotactic measurement and analysis using a 3D digitizer is a useful and accurate method for the clinical anatomy study of neurovascular structure.

## Methods

### Cadaveric dissection

All procedures were performed in accordance with the Declaration of Helsinki of the World Medical Association (WMA). After the approval of the Surgical Anatomy Education Centre, Yonsei University College of Medicine, thirty embalmed cadavers that were legally donated to this institution, were subjected to the dissection of the back-neck and occipital region. Seventeen cadavers were male and 13 cadavers were female. The mean cadaver age was 80.2 years (50–101 years). Cadavers showing severe asymmetry with an abnormal position were excluded. While each body lay prone where EOP and the vertebra prominens were aligned horizontally, the skin and subcutaneous tissue on the neck and occipital region were removed. The back muscles, GON and 3ON, and OA were carefully exposed by meticulous dissection. With pins, the nerves and artery were fixed to prevent unintentional displacement. Dissection was performed by exposures of the trapezius, SpC, SSC, and suboccipital triangle.

### Stereotactic measurement with the 3D digitizer

The locations of the piercing points of the two cutaneous nerves at every layer were recorded using photographs and notes. Thereafter, the 3D coordinates of points were recorded with a 3D digitizer. The coordinates were saved in a Microsoft Excel file (2013 version; Microsoft Corp., Redmond, Washington, USA). Before taking the location of the points, four landmark points (EOP, spinous process tip of the seventh cervical vertebra, and the lowermost point of the mastoid process of both sides) were taken. The following points and plane were determined;

1. EOP
2. Lowermost point of the mastoid process (right and left sides; MPR and MPL)
3. Most posterior tip of the spinous process of the seventh cervical vertebra (vertebra prominens; CV7)
4. Midplane (a plane through EOP, lowermost point of the mastoid process and midpoint of the MPR and MPL)
5. Horizontal plane (a plane perpendicular to the midplane and through the EOP)
6. Most right and left points of the SNL
7. Emerging points of GON from the trapezius and SSC
8. Emerging point of GON from OCI after looping this muscle
9. Emerging points of 3ON from the trapezius, SpC, and SSC.

### Data analysis

Regarding the coordinates of points measured by the 3D digitizer, we standardised the coordinate system using two steps. First, the coordinate of each point $$(x,y,z)$$ was translated into $$(x^{\prime} ,y^{\prime} ,z^{\prime} )=$$$$(x-{x}_{EOP},y-{y}_{EOP},z-{z}_{EOP})$$, so that new coordinates of EOP were set to the origin. Thereafter, linear transformation, which preserves the scale of the coordinate system, was performed to satisfy the following criteria:

1. The y-axis should be along the line passing EOP (the origin) and CV7, so that the final coordinate of CV7 is transformed into $({x}_{CV7}^{\prime\prime} ,{y}_{CV7}^{\prime\prime} ,{z}_{CV7}^{\prime\prime} )=(0,{d}_{EOP\_CV7},0)$, where ${d}_{EOP\_CV7}$ denotes the distance between EOP and CV7 in the original coordinates.
2. The x-axis should be along the SNL lines, so the coordinates of most right and left points of SNL, and the midpoint between them are transformed into $({d}_{SNLR\_SNLL}/2,0,{d}_{EOP\_SNL})$, $(-{d}_{SNLR\_SNLL}/2,0,{d}_{EOP\_SNL})$, and $(0,0,{d}_{EOP\_SNL})$ respectively.
3. The x-value of the midpoint (MP) of MPR and MPL should be zero, so the transformed coordinate of MP should be $(0,{d}_{EOP\_MP}\,\cos \,\theta ,{d}_{EOP\_MP}\,\sin \,\theta )$ where $\theta$ is the angle between the line passing EOP and CV7 (y-axis of the final coordinate system), and the line passing EOP and MP. This angle can be calculated by the inner product of two vectors.
4. The coordinates of MPR and MPL should be $({d}_{MPR\_MPL}/2,{d}_{EOP\_MP}\,\cos \,\theta ,{d}_{EOP\_MP}\,\sin \,\theta )$ and $({d}_{MPR\_MPL}/2,{d}_{EOP\_MP}\,\cos \,\theta ,{d}_{EOP\_MP}\,\sin \,\theta )$, respectively.

These criteria consist of rules about transformations of seven points. At least, three conditions are required to determine linear transformation in the 3D system. However, in this case, the number of conditions to be fulfilled is greater than three (mathematically “overdetermined”), so the matrix of this transformation was identified using a pseudo-inverse matrix (also known as the least-square solution). After we determined the transformation matrix, all the coordinates we measured were standardised by the matrix. We implemented these procedures as a program code using R 3.3.2, so the standardization was carried out automatically.

After the transformation, locations of the emerging points of GON on the trapezius, SSC, and OCI were established as depth (distance from the horizontal plane), horizontal position (distance from the midplane), and vertical position (distance from EOP), respectively; i.e. z, y, and x-values in the standardised coordinate system, respectively. The locations of the emerging points of 3ON on the trapezius, SpC, and SSC were established in the same manner. The data are presented as mean ± SD values. After the removal of all muscles over the suboccipital triangle, the overall courses of two cutaneous nerves was observed.

In addition, we obtained 3D images of the back muscles and adjacent skeleton and reconstructed our coordinates of average value based on this image. This reconstructed stereotactic image is provided as supplementary data in this article.

## Electronic supplementary material

Supplementary Information

## References

[1] Pingree MJ, Sole JS, OʼBrien TG, Eldrige JS, Moeschler SM (2017). Clinical Efficacy of an Ultrasound-Guided GON Block at the Level of C2. Reg Anesth Pain Med.

[2] Mosser, S. W., Guyuron, B., Janis, J. E. & Rohrich, R. J. The anatomy of the GON: implications for the etiology of migraine headaches. *Plast Reconstr Surg* **113**, 693-7-700 (2004). 10.1097/01.PRS.0000101502.22727.5D 14758238

[3] Bogduk N (1982). Clinical Anatomy of the Cervical Dorsal Rami. Spine (Phila. Pa. 1976)..

[4] Choi I, Jeon SR (2016). Neuralgias of the head: Occipital neuralgia. J Korean Med Sci.

[5] Janis, J. E. e*t al*. The Anatomy of the Greater Occipital Nerve: Part II. Compression Point Topography. *Plast Reconstr Surg* **126**, 1563–1572 (2010). 10.1097/PRS.0b013e3181ef7f0c 20639804

[6] Janis JE (2010). Neurovascular Compression of the Greater Occipital Nerve: Implications for Migraine Headaches. Plast Reconstr Surg.

[7] Natsis K (2006). The course of the GON in the suboccipital region: A proposal for setting landmarks for local anesthesia in patients with occipital neuralgia. Clin Anat.

[8] Cohen SP (2012). Headaches during war: Analysis of presentation, treatment, and factors associated with outcome. Cephalalgia.

[9] Magńsson T, Ragnarsson T, Björnsson A (1996). Occipital nerve release in patients with whiplash trauma and occipital neuralgia. Headache.

[10] International Headache Society. The International Classification of Headache Disorders: 2nd edition. *Cephalalgia* **24****Suppl 1**, 9–160 (2004). 10.1111/j.1468-2982.2003.00824.x 14979299

[11] Li R (2017). Single injection of a novel nerve growth factor coacervate improves structural and functional regeneration after sciatic nerve injury in adult rats. Exp Neurol.

[12] Lauretti GR, Corrêa SWRO, Mattos AL (2015). Efficacy of the GON Block for Cervicogenic Headache: Comparing Classical and Subcompartmental Techniques. Pain Pract.

[13] Naja ZM, El-Rajab M, Al-Tannir MA, Ziade FM, Tawfik OM (2006). Occipital nerve blockade for cervicogenic headache: a double-blind randomized. Pain Pr.

[14] Naja ZM, El-rajab M, Al-tannir MA, Ziade FM, Tawfik OM (2006). Repetitive occipital nerve blockade for cervicogenic headache: Expanded case report of 47 adults. Pain Pract.

[15] Skaribas I, Alo K (2010). Ultrasound imaging and occipital nerve stimulation. Neuromodulation.

[16] Na SH (2010). Ultrasonic doppler flowmeter-guided occipital nerve block. Korean J Anesthesiol.

[17] Greher M, Moriggl B, Curatolo M, Kirchmair L, Eichenberger U (2010). Sonographic visualization and ultrasound-guided blockade of the GON: A comparison of two selective techniques confirmed by anatomical dissection. Br J Anaesth.

[18] S Standring, Gray’s Anatomy 40th edition. *Churchill Livingstone* (2009).

[19] Bovim G (1991). Topographic variations in the peripheral course of the GON. Autopsy study with clinical correlations. Spine (Phila. Pa. 1976)..

[20] Koopman JSHA (2009). Incidence of facial pain in the general population. Pain.

[21] Watanabe K, Saga T, Iwanaga J, Tabira Y, Yamaki KI (2017). An anatomical study of the transversus nuchae muscle: Application to better understanding occipital neuralgia. Clin Anat.

[22] Kuhn WF, Kuhn SC, Gilberstadt H (1997). Occipital neuralgias: clinical recognition of a complicated headache. A case series and literature review. J Orofac Pain.

[23] Anthony, M. Headache and the GON. *Clin Neurol Neurosurg* **94** (1992). 10.1016/0303-8467(92)90177-5 1335856

[24] Dash, K. S., Janis, J. E. & Guyuron, B. The lesser and 3ONs and migraine headaches. *Plast Reconstr Sur*g **115**, 1752-1758-1760 (2005). 10.1097/01.prs.0000161679.26890.ee 15861086

[25] Becser N, Bovim G, Sjaastad O (1998). Extracranial nerves in the posterior part of the head. Anatomic variations and their possible clinical significance. Spine (Phila. Pa. 1976)..

[26] Kang H, Tian L, Mikesh M, Lichtman JW, Thompson WJ (2014). Terminal Schwann Cells Participate in Neuromuscular Synapse Remodeling during Reinnervation following Nerve Injury. J Neurosci.

[27] Ashkenazi, A. & Levin, M. Three common neuralgias: How to manage trigeminal, occipital, and postherpetic pain. *Postgrad Med* **116**, 16–24 + 31–32 + 48 (2004). 10.3810/pgm.2004.09.1579 15460087

[28] Loukas M (2006). Identification of greater occipital neve landmarks for the treatment of occipital neuralgia. Folia Morphol (Warsz).

[29] Menéndez LP (2017). Comparing Methods to Assess Intraobserver Measurement Error of 3D Craniofacial Landmarks Using Geometric Morphometrics Through a Digitizer Arm. J Forensic Sci.

[30] Alobaidy MA, Soames RW (2016). Evaluation of the coracoid and coracoacromial arch geometry on Thiel-embalmed cadavers using the three-dimensional MicroScribe digitizer. J Shoulder Elb Surg.

[31] Owaydhah WH, Alobaidy MA, Alraddadi AS, Soames RW (2017). Three-dimensional analysis of the proximal humeral and glenoid geometry using MicroScribe 3D digitizer. Surg Radiol Anat.

[32] Mottini M (2017). Stereographic measurement of orbital volume, a digital reproducible evaluation method. Br J Ophthalmol.

