# Supplementary material for: Stereotactic topography of the greater and third occipital nerves and its clinical implication
Source: Sci Rep. 2018 Jan 17;8:870. doi: 10.1038/s41598-018-19249-6 (PMC5772481; doi:10.1038/s41598-018-19249-6)
Supplement: Supplementary file 1 — Supplementary Information [file 41598_2018_19249_MOESM1_ESM.pdf]

Stereotactic topography of the greater and third occipital nerves and its clinical implication

Hong-San Kim[1#], Kang-Jae Shin[1#], Jehoon O[1], Hyun Jin Kwon[1], Minho Lee[2*]

and Hun-Mu Yang[1*]

# co-1st authors, *co-corresponding authors

[1]Department of Anatomy, Yonsei University College of Medicine, Seoul, Korea

[2]Catholic Precision Medicine Research Center, College of Medicine, The Catholic University of Korea, Seoul, Korea

Correspondence to:

Hun-Mu Yang, DDS, PhD

Department of Anatomy, Yonsei University College of Medicine

Address: 50-1 Yonsei-ro, Seodaemun-gu, Seoul, 03722, South Korea

Telephone: +82-2-2228-1649

E-mail: yanghm@yuhs.ac

Minho Lee, PhD

Catholic Precision Medicine Research Center, College of Medicine,

The Catholic University of Korea

Address: 222 Banpo-daero, Seocho-gu, Seoul, 06591, South Korea

Telephone: +82-2-2258-7584

E-mail: MinhoLee@catholic.ac.kr

Authors information

1. Hong-San Kim, PhD

Department of Anatomy, Yonsei University College of Medicine

Address: 50-1 Yonsei-ro, Seodaemun-gu, Seoul 03722, South Korea

Telephone: +82-2-2228-1648

E-mail: hongsan@yuhs.ac

2. Kang-Jae Shin, PhD

Department of Anatomy, Yonsei University College of Medicine

Address: 50-1 Yonsei-ro, Seodaemun-gu, Seoul 03722, South Korea

Telephone: +82-2-2228-1648

E-mail: SHINKJ@yuhs.ac

3. Jehoon O, BS

Department of Anatomy, Yonsei University College of Medicine

Address: 50-1 Yonsei-ro, Seodaemun-gu, Seoul 03722, South Korea

Telephone: +82-2-2228-1648

E-mail: jhoon81@naver.com

4. Hyun Jin Kwon, BS

Department of Anatomy, Yonsei University College of Medicine

Address: 50-1 Yonsei-ro, Seodaemun-gu, Seoul 03722, South Korea

E-mail: hjkwon128@yuhs.ac

Telephone: +82-2-2228-1648

5. Minho Lee, PhD

Catholic Precision Medicine Research Center, College of Medicine,

The Catholic University of Korea

Address: 222 Banpo-daero, Seocho-gu, Seoul, 06591, South Korea

Telephone: +82-2-2258-7584

E-mail: MinhoLee@catholic.ac.kr

6. Hun-Mu Yang, DDS, PhD

Department of Anatomy, Yonsei University College of Medicine

Address: 50-1 Yonsei-ro, Seodaemun-gu, Seoul, 03722, South Korea

Telephone: +82-2-2228-1649

E-mail: yanghm@yuhs.ac

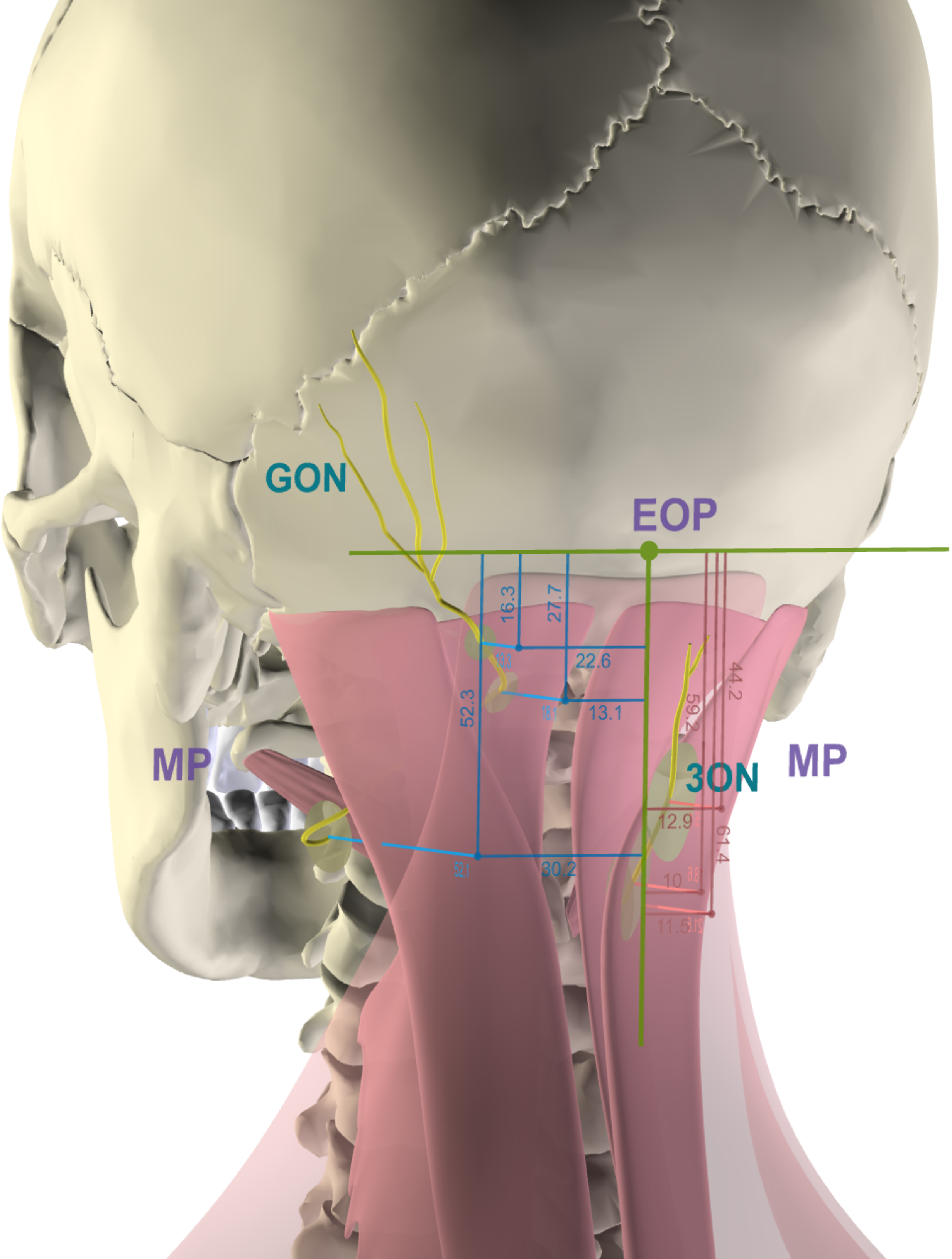
GON
EOP
MP
3ON
MP
16.3
27.7
22.6
13.1
52.3
30.2
44.2
59.2
12.9
61.4
10
11.5
